# Safety and costs of blood transfusion practices in dengue cases in Brazil

**DOI:** 10.1371/journal.pone.0219287

**Published:** 2019-07-08

**Authors:** Alessandra Aparecida Vieira Machado, Fábio Juliano Negrão, Júlio Croda, Elias Silva de Medeiros, Maria Aparecida dos Santos Pires

**Affiliations:** 1 Health Sciences College, Federal University of Grande Dourados, Dourados, Mato Grosso do Sul, Brazil; 2 Universitary Hospital of Federal University of Grande Dourados, Federal University of Grande Dourados, Dourados, Mato Grosso do Sul, Brazil; 3 School of Medicine, Federal University of Mato Grosso do Sul, Campo Grande, Mato Grosso do Sul, Brazil; 4 Oswaldo Cruz Foundation, Campo Grande, Mato Grosso do Sul, Brazil; 5 Faculty of Exact Sciences and Technology, Federal University of Grande Dourados, Dourados, Mato Grosso do Sul, Brazil; Vita Salute University of Milan, ITALY

## Abstract

**Background:**

Dengue is a public health problem, and noncompliance with World Health Organization (WHO) recommendations for blood transfusion components is frequently reported. Moreover, economic impact studies of the WHO recommendations on the use of blood transfusion are scarce.

**Methods:**

We compared the cost and hospitalization time in a prospective observational study, by following hospitalised patients and analysing their medical records from 2010 and March 2016 to December 2017. We divided the patients into two groups: transfused (with or without WHO criteria for transfusion) and not transfused (with or without WHO criteria for transfusion). Generalised linear modelling was performed to identify the variable that could increase the costs and hospital stay.

**Results:**

Among 323 patients, 52 were transfused, of whom 52% without criteria (n = 27), and 271 were not transfused, of which 4.4% (n = 12) with criteria. Hospitalisation costs were 41% higher in the transfused group without criteria than in those with criteria (median US$ 674.3 vs US$ 478 p = 0.293). Patients who were not transfused but met the WHO criteria for transfusion (n = 12) had longer mean hospitalisation time than did those who were not transfused (3.8±3.4 days versus 3.6±3.1 days; p = 0.022). The GLM analysis using hospital stay and costs as the dependent variable explained approximately 33.4% (R^2^ = 0.334) of the hospitalisation time and 79.3% (R^2^ = 0.793) of costs. Receiving a transfusion increased the hospitalization time by 1.29 days (p = 0.0007; IRR = 1.29), and the costs were 5.1 times higher than those without receiving blood components (IRR = 5.1; p< 0.001; median US$ 504.4 vs US$ 170.7). In contrast, patients who were transfused according to WHO criteria had a reduction in costs of approximately 96% (IRR = 0.044; p<0.001; β = -3.12) compared to that for those who were not transfused according to WHO criteria (without criteria).

**Conclusion:**

Transfusion without following WHO recommendations increased the time and cost of hospitalisation.

## Introduction

Dengue is a vector-borne viral infection and an important public health concern in tropical and subtropical regions worldwide. Before 1970, nine countries experienced severe dengue epidemics. Today, the disease is endemic in more than 100 countries in the World Health Organization (WHO) African, American, Eastern Mediterranean, South-East Asian and Western Pacific regions; the American, South-East Asian and Western Pacific regions are the most seriously affected [1]. The actual numbers of dengue cases are underreported, and many cases are misclassified. However, one recent estimate indicates that 390 million dengue infections occur every year; among these infections, 96 million manifest clinically (with any degree of disease severity) [2]. An estimated 3.9 billion people in 128 countries are at risk of infection with dengue virus [3], and approximately 500,000 cases require hospitalisation [1].

Dengue has a wide spectrum of clinical presentations, often with unpredictable clinical evolution and outcomes. The infection causes flu-like illness (dengue without warning signs—DWWS) and occasionally develops into a potentially lethal complication called severe dengue (SD) [1, 4]. Thrombocytopenia is commonly reported because immunological peripheral destruction of platelets and decreased platelet production due to bone marrow suppression and attenuation of megakaryocyte maturation occur in dengue fever; however, dengue thrombocytopenia is transient, and spontaneous platelet count recovery is always observed [5].

According the WHO, the use of blood components can be exceptionally therapeutic, but the use of blood components, especially platelets, should be limited to 22–50% of adult patients in various settings; additionally, blood transfusion in dengue is polemic and about 22–23% of are considered inappropriate [6, 7]. Thus, research that contributes to patient safety programmes are necessary to implement measures to reduce risks and mitigate side effects [8]. The economic impact studies of inappropriate blood components transfusions in dengue is scarce, and the urgency of these studies to fill information gap is clear [9, 10].

Dengue has been associated with substantial cost to the healthcare sector and national economy in endemic countries; if control strategies could reduce dengue appreciably, billions of dollars could be saved globally [11–14].

Practices not recommended by the WHO increase the associated costs [15]. Nevertheless, recent economic literature regarding the transfusion of blood components is scarce and does not include any research that describes the economic impact of not following WHO guidelines for the transfusion of blood components (platelets, fresh frozen plasma–FFP, and packed red cells–PRC). The aim of this study was to describe the use of blood components in clinical practice and to assess its economic and patient health impact through hospitalised dengue cases generated by adherence or a lack of adherence to WHO recommendations.

## Methods

### Study site and design

This was a prospective observational study, conducted in two stages in Dourados City, which is located in Mato Grosso do Sul (MS) State in Midwest Brazil. Dourados is the second largest city in MS State, has an estimated population of 218,069 inhabitants [16] and is a health reference centre for 30 other surrounding cities. Additionally, this city has economic and political importance in the region.

The first stage included all dengue cases hospitalised from January to December 2010 during the largest dengue epidemic in the city of the last 8 years, and the second stage was conducted from March 2016 to December 2017 during the non-epidemic year. All patients included, were followed during all the time of hospitalization. In all cases, responses to a questionnaire were provided and primary medical records were analysed.

At both stages, all cases with clinically and laboratory-confirmed dengue using non-structural protein 1 (NS1), IgM antibody capture enzyme-linked immunosorbent assay (MAC-ELISA) and/or reverse transcription polymerase chain reaction (RT-PCR) according to the manufacturer’s instructions [17, 18]. The dengue cases included in our study were reported to the official database or the National System for Reportable Diseases (Sistema Nacional de Agravos de Notificação–SINAN).

Clinically suspected dengue was defined in accordance with the WHO as febrile illness with at least two or more of the following manifestations: headache, retro-orbital pain, myalgia, joint pain, rash or any bleeding symptoms. Next, a local team of epidemiologists conducted an investigation of each notification to confirm or exclude each case using WHO inclusion criteria [19].

A bottom-up approach was used [20, 21] to determine the direct medical costs of the hospitalisation of dengue cases using the Health System Agency Funding perspective. The costs of each hospital stay were obtained directly from hospital records [22, 23].

### Inclusion and exclusion criteria

The laboratory-confirmed dengue cases reported to SINAN during the study period hospitalised in the participating hospitals in this study, regardless of age, sex, race and clinical presentation were included. We excluded cases that received a modified diagnosis that excluded dengue virus infection. Confirmed dengue cases were defined as patients exhibiting symptoms and/or clinical signs such as febrile illness with at least one clinical manifestation suggestive of dengue illness, including headache, retro-orbital pain, myalgia, joint pain, rash or any bleeding symptoms with laboratorial confirmation [1].

### Criteria for the use of blood components

There is no agreement about the use of blood or blood component to dengue clinical management [24]. However, in this research the WHO/PAHO recommendations for the use of blood components are the criteria used for transfusion. Table 1 summarizes the recommendations [1, 25]. The Brazilian Ministry of Health uses the same recommendations for Severe Dengue treatment [26].

**Table 1:** WHO recommendations for the use of blood components.

Blood component	WHO Recommendations
Platelet	Persistent uncontrolled bleeding after a state of shock, with corrected coagulation factors and with thrombocytopenia and prolonged bleeding time 1.5 times higher than normal.
	Caesarean section or other emergency surgery with risk of bleeding, the platelet count should be >50,000 mm^3^; in eye surgery and neurosurgery, the platelet count should be >100,000 mm^3^.
Packed Red Cell (PRC)	Blood transfusion should be given as soon as severe bleeding is suspected or recognised;
	Sudden drop in haematocrit is not accompanied by patient improvement; these findings indicate possible major bleeding and the need to cross-match and transfuse packed red blood cells (5 to 10 mL/kg).
Fresh Frozen Plasma (FFP)	Fibrinogen>100 mg and prothrombin time (PT) and activated partial thromboplastin time (aPTT) are more than 1.5 times the standard reference values, consider transfusion of frozen fresh plasma (10 ml/kg) within 30 minutes.

Patients who met the criteria described in Table 1 for transfusion were classified as "with criteria"; patients who did not meet those criteria were classified as "without criteria”.

### Data collection

Data sets were collected through the analysis of medical records from hospitalised patients with confirmed diagnoses of dengue at selected hospitals. A structured questionnaire (S1 Table) were applied for socio-demographic characterisation (age, sex, race, and schooling), epidemiologic characterisation (signs and symptoms, the results of exams, the presence of comorbidities, the use of blood components, with or without fitting the criteria recommended by the WHO, final classification of case and patient outcome (cure or death)). In addition, we obtained financial variables (type of hospitalisation, regular ward (RW) or intensive care unit (ICU), charges for medications and hospital supplies, examinations, daily allowances and fees).

All costs collected in 2010 were calculated in the local currency (real) and adjusted for inflation during the period from December 2010 to July 2017 before being converted to US dollars (US$) using the exchange rate (R$ 1 = US$ 0.3206) of the Brazilian Central Bank on July 31, 2017 (http://www.bcb.gov.br). The 2016–2017 costs were not necessarily adjusted for inflation.

### Data analysis

The double typing of questionnaires was performed using EpiData version 3.1 (Lauristen JM (Ed.), Odense, Denmark), SAS 9.1 (SAS Institute, Cary, NC) and R software (R Core Team, 2018) for statistical analyses. Categorical variables are expressed as proportions, and continuous variables are expressed as the means and standard deviations or medians and interquartile ranges (IQRs; 75^th^ and 25^th^). Kolmogorov-Smirnov and Shapiro-Wilk tests were used to verify that the data were normally distributed. We used non-parametric tests, such as the Mann-Whitney U-test, to compare the medians, and the chi-squared test to compare the proportions, with a significance level of 5% (p<0.05).

To identify the variable that could increase the costs and hospital stay, generalised linear modelling (GLM) was performed. For the variable ‘time’, a Poisson GLM was used and for the variable ‘costs’, a Gaussian GLM was used, both with a logarithmic link function.

Stepwise analysis with the Akaike information criterion (AIC) for selection was used to choose a better model. The independent variables tested were sex, dengue classification, age, time of hospitalisation, type of hospitalisation such as ICU or RW or ICU plus RW, comorbidities, side effects by blood components and criteria for using blood components. The significance level was set at p≤0.05.

### Ethical considerations

The study was approved by the Human Research Ethics Committee of the Federal University of Grand Dourados (UFGD-protocol numbers 003/2011 and 1.481.062/2016) and was registered at the Brazilian Ethical Office (Plataforma Brasil: http://plataformabrasil.saude.gov.br/visao/pesquisador/gerirPesquisa/gerirPesquisaAgrupador.jsf), and was conducted in accordance with all relevant tenets of the World Medical Association’s Declaration of Helsinki. All participants provided written consent before study entry and their confidentiality was ensured during data collection by replacing names with alphanumeric codes.

## Results

**Fig 1:**
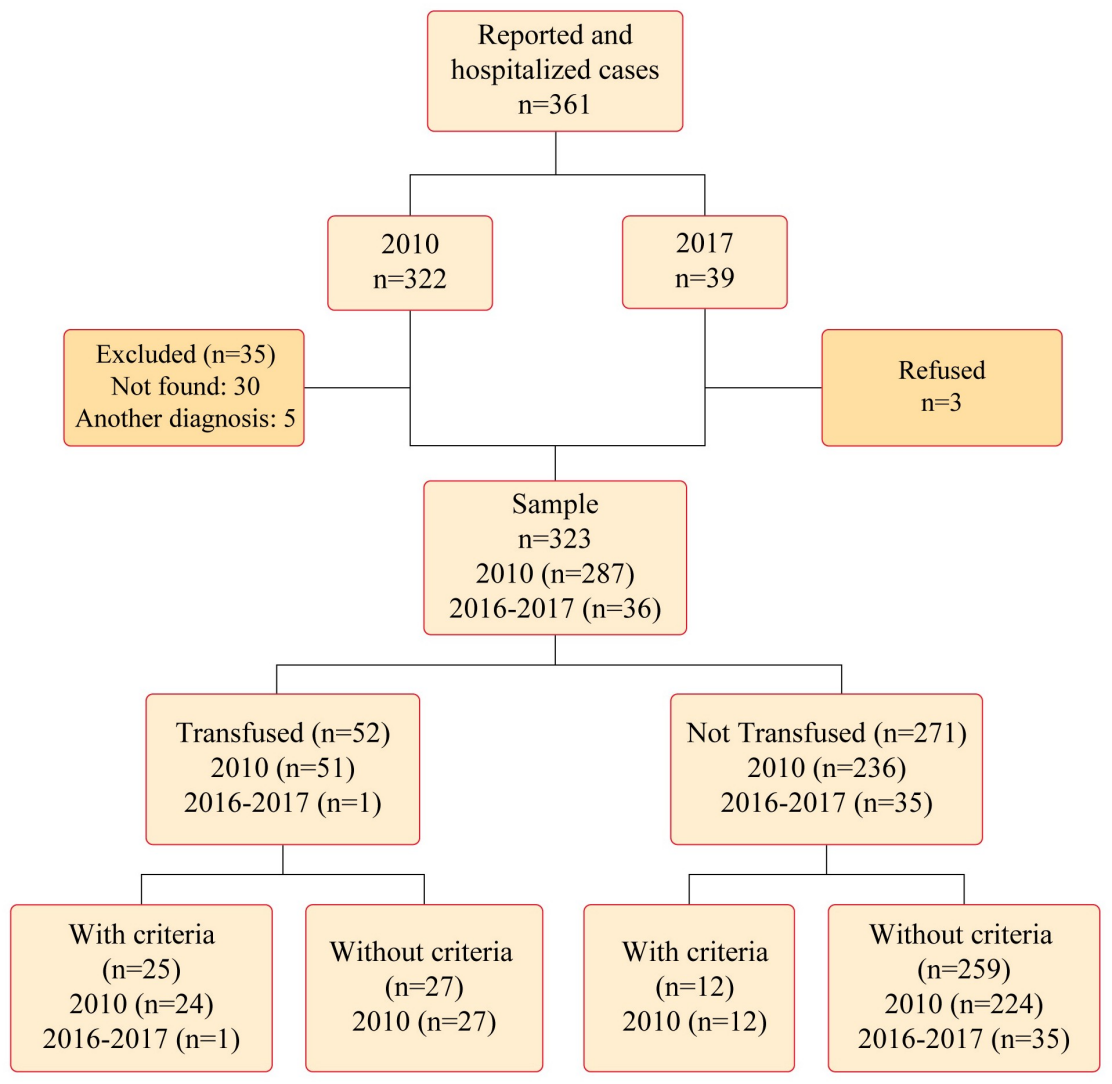
Flowchart of the selection and sampling.

From January 1^st^, 2010, to December 31^st^, 2010, and from March 1^st^, 2016, to December 31^st^, 2017, we assessed 361 eligible patients. Among these patients, the following 323 were included: 287 hospitalised (56.8% of all hospitalised cases in the city) in 2010 and 36 (97% of all hospitalised cases in the city) in 2016–2017. Among these 323 patients, 52 (16.1%) received transfusions of blood components; 25 (48%) received transfusions according to WHO recommendations (with criteria), and 27 (52%) received transfusions without criteria according to WHO recommendations. Among 271 patients that were not transfused, 12 should have received blood components according to WHO guidelines (with criteria). Fig 1 describes the sampling protocol of this study.

The transfusion group received platelet transfusion (n = 44), PRC transfusion (n = 03), FFP transfusion (n = 02), platelet plus PRC transfusion (n = 02) and platelet plus FFP transfusion (n = 1). The majority of transfusions (98%) occurred in 2010. The main laboratory and clinical indications are shown in Table 2.

**Table 2:** Laboratory and clinical characteristics of transfused and non-transfused patients.

	Transfused (n = 52)		Not transfused (n = 271)	
	With criteria (n = 25)	Without criteria (n = 27)	With criteria (n = 12)	Without criteria (n = 259)
**Platelets**				
≤20,000 with bleeding	15	0	11	0
≤20,000 without bleeding	0	10	0	58
20,000–50,0000 with bleeding	3	0	1	0
20,000–50,000 without bleeding	0	10	0	76
>50,000	1	5	0	125
**Red Blood Cells**				
Hb ≤ 7 g%	2	0	0	0
Hb >7 g% with bleeding	1	0	0	0
**Plasma + Platelets**				
Haemorrhage with haemodynamic decompensation and platelets >20,000	1	0	0	0
**Platelets + Red Blood Cells**				
Haemorrhage with haemodynamic decompensation and platelets >54,000	2	0	0	0
**Plasma**				
Normal TTPA, no bleeding	0	2	0	0

Characteristics such as age and comorbidities were higher in the transfused group (with criteria) but were nonsignificant (p = 0.562). However, in the non-transfused group (with criteria), the adult population (15 to 59 years old) appeared to have a greater need for transfusion (p = 0.025) than did children or the elderly. In addition, the presence of comorbidities was higher in the non-transfused group with criteria but was nonsignificant (p = 0.516) (Table 3).

**Table 3:** Demographic and hospitalisation characteristics of study population (n = 323).

	Transfused (n = 52)				X^2^	Not Transfused (n = 271)				X^2^
	with criteria		without criteria			with criteria		without criteria		
	(n = 25)		(n = 27)			(n = 12)		(n = 259)		
	n	%	N	%		N	%	N	%	
**Sex**										
Male	11	44	10	37	0.421	6	50	95	36.7	0.297
Female	14	56	17	63		6	50	164	63.3	
**Age**										
< 15	4	16	5	18.5	0.562	0	0	54	20.9	0.025
15–60	12	48	19	70.4		9	75	150	57.9	
≥60	9	36	3	11.1		3	25	55	21.2	
**Dengue classification**										
Without warning signs	0	0	14	51.9	0.394	0	0	152	58.7	0.140
With warning signs	10	40	6	22.2		11	91.7	96	37.1	
Severe	15	60	7	25.9		1	8.3	11	4.2	
**Comorbidities**										
Yes	12	48	14	51.9	0.523	7	58.3	104	40.2	0.516
No	12	48	13	48.1		2	16.7	137	52.9	
Unknown	1	4	0	0		3	25	18	6.9	
**Type of hospitalisation**										
Regular Ward	15	60	21	77.8	0.048	10	83.3	248	95.8	0.371
Intensive care unit (ICU)	3	12	0	0		0	0	5	1.9	
Regular ward and ICU	7	28	6	22.2		2	16.7	6	2.3	
**Adverse events by blood components**										
Yes	1	4	3	11.1	0.337	-	-	-	-	-
No	24	96	24	88.9						
Urticarial rash	1	4	3	11						
Chest pain	0	-	1	3.7						
Hypothermia	1	4	2	7.4						
**Hospitalisation time**										
Median (IQR)	5(6–4)			2(4–1)	0.107*	3 (5–2)		3 (4–2)		0.023*
mean (± SD)	6.8(±7.4)			2.7 (±1.7)		3.8(±3.4)		3.6 (±3.1)		
**Case outcome**										
Recovery	21	84	27	100	0.048	12	100	259	100	0.3711
Death	4	16	0	0		0	0	0	0	

* Mann Whitney test-U-test

Between transfused patients with and without criteria for transfusion, SD was more frequent in patients with criteria (15/25; 60%) than in those without criteria (7/27; 25.9%). Furthermore, in these groups, staying in the ICU or RW plus ICU was more frequent in transfused patients with criteria than in those transfused without criteria (p = 0.048). Additionally, transfusion side effects occurred in 4 patients (7.7%), 3 in the group without criteria and 1 in the group with criteria. These side effects included hypothermia plus urticarial rash (n = 2), urticarial rash plus chest pain (n = 1) and chest pain (n = 1). No death was reported in association with side effects (Table 3).

All cases of death occurred among the patients who underwent transfusion with criteria (p = 0.048). We could not show an association with the use of blood components; however, these transfused patients had exacerbating comorbidities, including three with type II diabetes, hypertensive and older age and one with liver cirrhosis. The final patient was 47 years old and had hypertension, diabetes, and systemic lupus erythematosus.

Regarding the hospital stay and costs, although nonsignificant (p = 0.107), the length of hospitalisation was higher in patients transfused with criteria. However, when we analysed the cost, these values were lower in patients transfused with criteria (median US$ 478.0) than in patients transfused without criteria (median US$ 674.3) (p = 299). In the non-transfused patient group (with criteria vs. without criteria), both time (p = 0.022) and cost (p = 0.225) were higher in non-transfused patients with WHO criteria for transfusion (Table 4).

**Table 4:** Comparison of hospitalisation time and stratified costs for transfusion in patients with or without the WHO criteria (n = 323).

Variable	Transfused (n = 52)				Not Transfused (n = 271)			
	With criteria (n = 25)	Without criteria (n = 27)	U-test	All	With criteria (n = 12)	Without criteria (n = 259)	U-test	All
**Costs (US$)**								
Median	478	674.3	0.293	504.4	220.4	164.4	0.225	170.7
(IQR)	1240.2–295.2	1072–403.8		1096.9–369.6	571–137.5	403.4–136.9		404.3–136.9
Mean	1220.3	1644.5		1440.5	675	366.2		379.8
(±SD)	2676.5	3190		2933.2	1008	604.2		627.9
**Time (days)**								
Median	5	2	0.107	6	4	3	0.022	3
(IQR)	(6–4)	(4–1)		(8–4)	(5–2)	(4–2)		(4–2)
Mean (±SD)	6.8 (±7.4)	2.7 (±1.7)		7 (±5.7)	3.8 (±3.4)	3.6 (±3.1)		3.7 (±3.1)

In the multivariate analysis model, we observed the best model that fit the assumptions and obtained a better AIC score (lower AIC). The highest adjusted R-squared (R^2^) using hospital stay (days) and costs as the dependent variable is shown in Table 5. These models explained approximately 33.4% (R^2^ = 0.334) of hospitalisation time and 79.3% (R^2^ = 0.793) of hospitalisation costs.

**Table 5:** Results of the generalised linear modelling (GLM) with hospital stay and cost as the dependent variable (n = 323).

Variables	Hospital Stay (days)				Costs (US$)			
	Β	Std. Error	*P*-value	IRR^a^	Β	Std. Error	*P*-value	IRR^a^
Intercept	1.509	0.095	<0.001	4.320	4.254	0.336	<0.001	70.41
Sex	*	*	*	*	*	*	*	*
Dengue Classification								
DWWS	-0.451	0.095	<0.001	0.637	-0.785	0.218	0.0003	0.456
DWS	-0.214	0.089	0.017	0.807	-1.508	0.229	<0.001	0.221
Criteria (yes)^b^	*	*	*	*	-3.120	0.204	<0.001	0.044
Comorbidities (yes)	0.141	0.055	0.010	1.151	*	*	*	*
Transfused (yes)	0.254	0.075	0.0007	1.290	1.626	0.151	<0.001	5.083
Age	*	*	*	*	0.041	0.003	<0.001	1.042
Type of hospitalisation								
Regular Ward	0.299	0.228	0.190	1.349	3.076	0.369	<0.001	21.672
ICU	-0.393	0.290	0.176	0.675	2.339	0.544	<0.001	10.371
Regular plus ICU	0.834	0.086	<0.001	2.303	1.010	0.087	<0.001	2.746
Time	-	-	-	-	0.129	0.009	<0.001	1.138

*Variables that did not remain in the final model, AIC criteria stepwise.

^a^ Incidence Rate Ratio (IRR) is transformed from the coefficient and is equal to exp (β).

^b^ Criteria (yes) means patients who met the criteria for transfusion

Regarding blood components, compared with not receiving transfusion, receiving transfusion (with or without criteria) increased the length of hospital stay by 1.29 days (p = 0.0007; IRR = 1.29) and increased the costs by 5.1 times (IRR = 5.1; p<0.001). In addition, patients with comorbidities stayed 1.2 days more than those without comorbidities. However, the variable ‘comorbidities’ was nonsignificant with regard to hospital costs.

Moreover, compared with transfusion without following WHO recommendations (without criteria), transfusion used according to WHO recommendations reduced costs by approximately 96% (IRR 0.044; p<0.001; β = -3.12). However, the variable ‘criteria’ was nonsignificant with regard to the increased duration of hospitalisation (Table 5).

In the cost evaluation by dengue disease WHO classifications, compared with patients with SD, patients with DWWS and dengue with warning signs (DWS) had an approximately 36.3% (p<0.001; IRR = 0.637) and 19.3% (p = 0.017; IRR = 0.807) reduction in the duration of hospital stay, respectively. Costs were also lower in less severe cases of dengue fever (Table 5).

## Discussion

The current study found that 98% (51/52) of transfusions occurred in 2010; among these transfusions, 51.9% (27/52) were inconsistent with WHO recommendations (thrombocytopenia without bleeding), and this percentage was higher than that found by *Pallavi* et al. (36.62%) [27], *Kumar* et al. (34.1%) [28] and others [7, 29, 30]. However, these authors examined the use of platelets only. In 2017, only one patient was transfused with criteria, demonstrating a considerable reduction in the use of blood components from 2010 to 2017.

We could not assess why the use of transfusion reduced from 2010 to 2017; a potential explanation is that 2010 was an epidemic year, whereas 2017 was not. Some authors have suggested that in a dengue epidemic, ‘dengue panic syndrome’ may exist in which physicians tend to overestimate the platelet count as the sole condition to indicate transfusion [7, 31, 32]. Alternatively, we hypothesised that these changes occurred due to increased physician compliance with WHO guidelines, which disseminated more evidence regarding the importance and safety of not using prophylactic platelets in dengue [11, 33–36].

Thrombocytopenia is common in dengue fever, but transient and spontaneous recovery in platelet counts is usually observed [28, 36, 37]. However, bleeding is a dreaded clinical manifestation of dengue fever. Thus, for years, prophylactic platelet transfusion has been performed in many cases in an attempt to reduce this risk. In recent years, several studies have demonstrated the importance of not using prophylactic blood components, especially platelets [7, 24, 28, 34–36, 38] because they were not superior to supportive care in preventing bleeding and had a higher incidence of side effects [11, 24, 37, 39].

The inappropriate use of blood components included platelets as well as Fresh Frozen Plasma (FFP). Two patients received FFP transfusion without criteria. To our knowledge, no studies have evaluated the use of FFP in cases of dengue; however, studies of other diseases have indicated that approximately 30–50% of FFP transfusions are prophylactic with or without a planned procedure, and a large proportion (up to 50%) of FFP transfusions do not follow guidelines [40–42].

Multivariate analysis showed that following the WHO recommendations resulted in a significant reduction in hospital costs (IRR = 0.044; p<0.001). Although the difference between median costs was nonsignificant (p = 0.293), patients transfused without WHO recommendations increased the direct median medical cost of hospitalisation. The average cost was approximately 41% higher (from US$ 478 to US$ 674.3), the equivalent of 53.7% to 75.8% of the median monthly Gross Domestic Product (GDP) (US$ 889.7) of the city population studied [43].

Costs were higher in cases transfused without criteria despite the shorter hospitalisation time, type of hospitalisation Intensive Care Unit (RW) or Regular Ward (RW) and dengue classification. Importantly, hospitalisations in the ICU and Severe Dengue (SD) are more expensive, and longer hospitalisation time results in greater expenses [12, 15, 44, 45]. However, in patients not transfused with criteria, hospitalisation time was higher than that in patients not transfused without criteria (p = 0.022).

Surprisingly, among the non-transfused group, 4.4% (12/271) should have received transfusions. Additionally, in the non-transfused group (not transfused with criteria), the median hospitalisation time (4 IQR 5–2) was significantly higher (p = 0.022) than that in the non-transfused group without criteria (3 IQR 4–2). Although nonsignificant, the median costs were 34.1% higher for not transfused with criteria (US$ 220.4 IQR 571–137.5) than for not transfused without criteria (US$ 164.4 IQR 403.4–136.9), demonstrating that not performing transfusion when indicated may compromise proper patient recovery and increase costs.

The selected multivariate model could explain 79.3% of costs and 33.4% of hospitalisation time. Variables such as living alone, previous infection by dengue, and the presence or absence of obesity could better explain factors influencing the costs and length of hospital stay [46, 47], but this information was unavailable.

Although the variable ‘criteria’ explained the increase in costs in GLM analyses (p<0.001), other variables, such as dengue classification, age, type of hospitalisation, and side effects, also contributed to increased costs. Therefore, the absence of WHO criteria for the use of blood components contributes to increased costs; however, this factor does not completely account for increased costs. The mean length of stay was similar to that in other studies performed with patients hospitalised due to dengue in Brazil [12, 15, 48].

No references were found to compare the hospitalisation time in cases of transfusion in Brazil for patients without WHO criteria. However, our result was similar to a study conducted by *Lye* et al. [49] in Singapore; in this study, patients who received platelet transfusions had a median hospital stay of 6 days (4–8 days), while patients who did not receive a transfusion had a median stay of 5 days (4–7).

Although only 4 of 52 patients (7%) experienced side effects, 75% (3/4) of these side effects could have been avoided as they resulted from transfusion in patients without criteria. In addition, the side effects occurred in a greater proportion of patients who were transfused without criteria (3/27; 11%) than that of those with criteria (1/25; 4%); however, this difference was nonsignificant (p = 0.3363). Moreover, the number of cases (n = 4) was too small to allow for a robust assertion that patients transfused without criteria had a higher likelihood of presenting side effects.

Blood components are expensive and potentially dangerous, and their availability is often limited [50]. Fresh Frozen Plasma (FFP) transfusions may cause allergic reactions with immediate hypersensitivity (e.g., urticaria and anaphylaxis) and a respiratory distress syndrome associated with pulmonary infiltrates, non-cardiogenic pulmonary oedema, which is mediated by soluble agents (cytokines). In addition, similar to other blood components, these transfusions carry the risk of infectious disease transmission, including hepatitis B and C, syphilis and human immunodeficiency virus (HIV) [40].

As described in detail previously [8], Patient safety is a fundamental principle of health care, and some countries have published studies showing that significant numbers of patients are harmed during health care, either resulting in permanent injury, increased length of stay in health care facilities, or even death [8, 51, 52]. Studies on direct medical costs associated with poor care show that additional hospitalisation, litigation costs, infections acquired in hospitals, lost income, disability and medical expenses have cost some countries between US$6 billion and US$29 billion per year [53].

Unfortunately, the design of the present study was limited; we could not evaluate the costs according to a societal perspective or determine why physicians did not follow the WHO recommendations, including why blood transfusions decreased from 2010 to 2017. Additionally, we could not determine whether the deaths were directly related to the use of blood products. However, this study analysed and quantified the costs of dengue cases that involved the use blood products, including platelets, FFP or Packed Red Cell (PRC), with or without adherence to WHO recommendations.

In conclusion, the use of blood components without criteria exposes patients to further risks, and adherence to WHO guidelines for the use of blood components reduces costs and hospitalisation times for patients hospitalised with dengue. We expect that our study will help policymakers and physicians raise awareness to promote patient safety.

## Supporting information

S1 Table Questionnaire. Questionnaire used for data collection.
(DOCX)
