# Supplementary material for: Safety and costs of blood transfusion practices in dengue cases in Brazil
Source: PLoS One. 2019 Jul 8;14(7):e0219287. doi: 10.1371/journal.pone.0219287 (PMC6613682; doi:10.1371/journal.pone.0219287)
Supplement: S1 Table — Questionnaire used for data collection. (DOCX) [file pone.0219287.s001.docx]

| 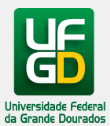RESEARCH - Characterization of dengue costs in Mato Gosso do Sul - Brazil | | | | | | | | | | |  |  |
| --- | --- | --- | --- | --- | --- | --- | --- | --- | --- | --- | --- | --- |
|  |  | |  |  |  |  |  |  |  |  |  |  |
|  |  | |  |  |  |  |  |  |  |  |  |  |
| Name and Surname: | | | |  |  |  |  |  |  |  |  |  |
| Identification Number: | | | | | |  |  |  |  |  |  |  |
|  |  | |  |  |  |  |  |  |  |  |  |  |
| TO PROTECT PATIENT CONFIDENTIALITY, THIS PAGE WILL BE SEPARATED AND REMOVED FROM THIS FORM BY THE COORDINATOR OR BY THE MAIN INTERVIEWER AFTER THE INTERVIEW AND BEFORE THE INFORMATION CONTAINED IN THIS FORM IS INCORPORATED IN A DATABASE. ALL FORMS THAT CORRESPOND TO THE SAME PATIENT MUST HAVE THE SAME IDENTIFICATION NUMBER SO THAT THE INFORMATION CAN BE MADE FROM DIFFERENT FORMS OF THE SAME PATIENT PARTICIPATING IN THE INVESTIGATION STUDY. | | | | | | | | | | |  |  |
| The objective of this study is to understand all relevant events that have occurred or are occurring with the patient and his / her relatives since the patient presented the first symptoms of this episode of illness. The patient or caregiver will give at least two interviews. The first interview will cover the period from the onset of the first symptoms of dengue until the time of the first interview. The second interview will cover the length of time from the first interview to the time of the second interview. The period covered by each interview is called the "Reference Period". | | | | | | | | | | |  |  |
|  |  | |  |  |  |  |  |  |  |  |  |  |
| **Phone:** | | | | | | | | | | |  |  |
|  |  | |  |  |  |  |  |  |  |  |  |  |
| **Email:** | | | | | | | | | | |  |  |
|  |  | |  |  |  |  |  |  |  |  |  |  |
| ----------------------------------------------------------------------------------------------------------------------------pull out here  before typing | | | | | | | | | | |  |  |
| **Q1** | | Identification Number | | | | | |  | Q1 |  |  |  |
| **Q2** | | It was a patient recruited (included in this study) as:  Not hospitalized 1- or 2- hospitalized | | | | | | | Q2 |  |  |  |
| **Q2-B** | | Health Service: 1- Public (governament) 2- Private | | | | | | | Q2-B |  |  |  |
| **Q2-C** | | Health care plan:  1- Unimed 2- Cassems 3- Particular  4- Cartão Saúde 5- PAX 6- CASSi  7- S. Bradesco 8- others 9-São Francisco | | | | | | | Q2-C |  |  |  |
| **Q3** | | Place of Recruitment (write the name of the health establishment): | | | | | | | Q3 |  |  |  |
| **Q4** | | Are you interviewing the patient or a responsible party? | | | | | | | Q4 |  |  |  |
|  | | 1- Patient | | 2- Responsible Party | | | 3- Both | |  |  |  |  |
| **Q5** | | If responsible party, indicate [degree of relatedness](https://www.linguee.pt/ingles-portugues/traducao/degree+of+relatedness.html). | | | | | |  | Q5 |  |  |  |
|  | | 1- Mother 2- Father 3- Grandfather 4- Grandmother  5- Older sibling 6- Other | | | | | | |  |  |  |  |
| **Q6** | | What is the location of the interview? | | | | | | | Q6 |  |  |  |
|  | | 1- Outpatient 2- Emergency Room 3- Hospitalized 4- Health Clinic/Office 5- Home 6- Other | | | | | | |  |  |  |  |
|  | |  |  |  |  |  |  |  |  |  |  |  |
| **Q7** | | Date of Interview n.1: | | | (mm/dd/yy) | |  |  | Q7 |  |  |  |
| **Q7-A** | | Date of Interview n.2: | | | (mm/dd/yy) | |  |  | Q7-A |  |  |  |
| **Q8** | | Birthdate: | | | (mm/dd/yy) | | |  | Q8 |  |  |  |
| **Q9** | | Age in years (completed): | | | |  |  |  | Q9 |  |  |  |
| **Q10** | | Sex: | 1- Male | |  | 2- Female | |  | Q10 |  |  |  |
| **Q11** | | Race / Color:  1-White 2- Black 3- Brown 4- Asian 9-Unreported | | | | | | | Q11 |  |  |  |
|  |  |  |  |  | |  | | |  |  |  |  |
| **Q12** | | Marital Status: | | | | | | | Q12 |  |  |  |
|  |  | 1- Married 2- Single 3- Common-law marriage 4- Separated  5- Widow(er) 9- Not Applicable | | | | | | |  |  |  |  |
| **Q13** | | What is the level of education:  1- Illiterate 2- Some Elementary/Middle School  3- Completed Middle School 4- Some High School  5- Completed High School 6- associate´s degree 7- Completed Undergraduate 8- Some undergraduate 9- Graduate school (master´s or doctorate) 00- Not applicable | | | | | | | Q13 |  |  |  |
|  |  |  | | |  |  | | |  |  |  |  |
| **Q14** | | How many years of school did the patient complete?  (total in years) | | | | | | | Q14 |  |  |  |
| **Q15** | | What is the patient's current type of employment?  1 – Public Service 2 – Employee 3 – Self-Employed 4 – Businessman/Employer 5 – Unemployed 9- Not applicable  10- Retired | | | | | | | Q15 |  |  |  |
|  | |  |  |  |  |  |  |  |  |  |  |  |
|  | |  |  |  |  |  |  |  |  |  |  |  |
|  | |  |  |  |  |  |  |  |  |  |  |  |
| ***ATTENTION: INFORMATION ABOUT THE RESPONSIBLE PARTY, complete for patients that are minors.*** | | | | | | | | | | |  |  |
| **Q15-A** | What is the responsible party's age (in years)? | | | | | | | | Q15-A |  |  |  |
| **Q15-B** | What is the reponsible party's level of education? | | | | | | | | Q15-B |  |  |  |
|  | 1- No formal education 2- Some Elementary/Middle School  3- Completed Middle School 4- Some High School  5- Completed High School 6- associate´s degree 7- Completed Undergraduate 8- Some undergraduate 9- Graduate school (master´s or doctorate) 00- Not applicable | | | | | | | |  |  |  |  |
|  |  |  |  |  |  |  |  |  |  |  |  |  |
|  |  |  |  |  |  |  |  |  |  |  |  |  |
|  |  |  |  |  |  |  |  |  |  |  |  |  |
|  |  |  |  |  |  |  |  |  |  |  |  |  |
| **Q16** | What is your average salary? | | | | | | | | Q16 |  |  |  |
|  | (Value rounded off in R$ - ex. 2000) | | | | | |  |  |  |  |  |  |
| **Epidemological variables / risk factors** | | | | | | | | | | |  |  |
| **Q17** | How many days since start of symptoms?  (indicate number of days) | | | | | | | | Q17 |  |  |  |
| **Q18** | Do you currently have any symptoms? 1- Yes 2- No | | | | | | | | Q18 |  |  |  |
| Which of the following signs and symptoms have you experienced during the reference period? | | | | | | | | | | |  |  |
| **Q19** | Vomiting? 1- Yes 2- No 3- No Unsure | | | | | | | | Q19 |  |  |  |
| **Q20** | Nausea?  1- Yes 2- No 3- No Unsure | | | | | | | | Q20 |  |  |  |
| **Q21** | Excessive thirst? 1- Yes 2- No 3- No Unsure | | | | | | | | Q21 |  |  |  |
| **Q22** | Diuresis reductions? 1- Yes 2- No 3- No Unsure | | | | | | | | Q22 |  |  |  |
| **Q23** | Headache?? | | | 1- Yes 2- No 3- No Unsure | | | | | Q23 |  |  |  |
| **Q24** | Petechias? | | | 1- Yes 2- No 3- No Unsure | | | | | Q24 |  |  |  |
| **Q25** | Eye pain? | | | 1- Yes 2- No 3- No Unsure | | | | | Q25 |  |  |  |
| **Q26** | Joint or muscular pain? 1- Yes 2- No 3- No Unsure | | | | | | | | Q26 |  |  |  |
| **Q27** | Any Bleeding? (small red spots on the skin, spontaneous or caused injuries ,nosebleeds, gum bleeds or any other type of bleeding) | | | | | | | | Q27 |  |  |  |
|  |  | |  |  |  |  |  |  |  |  |  |  |
|  |  | | 1- Yes 2- No 3- No Unsure | | | | | |  |  |  |  |
| **Q28** | Abdominal pain? 1- Yes 2- No 3- No Unsure | | | | | | | | Q28 |  |  |  |
| **Q29** | Diarrhea? | | | 1- Yes 2- No 3- No Unsure | | | | | Q29 |  |  |  |
| **Q30** | Sore throat or nasal secretions? 1- Yes 2- No 3- No Unsure | | | | | | | | Q30 |  |  |  |
| (Risk factors) Do you have any of the health issues below? | | | | | | | | | | |  |  |
| **Q32** | Diabete? | | | 1- Yes 2- No 3- No Unsure | | | | | Q32 |  |  |  |
| **Q33** | Systemic arterial hypertension?  1- Yes 2- No 3- No Unsure | | | | | | | | Q33 |  |  |  |
| **Q34** | Obesity? (BMI ≥30)? 1- Yes 2- No 3- No Unsure | | | | | | | | Q34 |  |  |  |
| **Q35** | Hepatopathies? ([liver cirrhosis](https://www.linguee.com.br/ingles-portugues/traducao/liver+cirrhosis.html) or others)  1- Yes 2- No 3- No Unsure | | | | | | | | Q35 |  |  |  |
| **Q36** | Allergies? | | | 1- Yes 2- No 3- No Unsure | | | | | Q36 |  |  |  |
| **Q37** | Cardiopathy? | | | 1- Yes 2- No 3- No Unsure | | | | | Q37 |  |  |  |
| **Q38** | Age Extremes? (>60 e <2 years old)? | | | | | 1- Yes 2- No 3- No Unsure | | | Q38 |  |  |  |
| **Q39** | Immunosuppresion? 1- Yes 2- No 3- No Unsure | | | | | | | | Q39 |  |  |  |
| **Q40** | Social Risk? *(living alone, unaccompanied elderly person, difficulty accessing the health center, transportation difficulties)* | | | | | | | | Q40 |  |  |  |
|  |  | |  | 1- Yes 2- No 3- No Unsure | | | | |  |  |  |  |
| **Q41** | Does the patient have any risk factors *(if any field between Q32 and Q 40 was marked as YES)* | | | | | | | | Q41 |  |  |  |
|  |  | |  |  |  |  |  |  |  |  |  |  |
|  |  | | 1- Yes | 2- No | 3- No Unsure | | 9- Ignored | |  |  |  |  |
| **Q42** | Dengue classification during the Reference Period? | | | | | | | | Q42 |  |  |  |
|  | 1- Dengue without warning signs 3- Severe Dengue | | | | | | | |  |  |  |  |
|  | 2- Dengue with warning signs 4- another diagnosis | | | | | | | |  |  |  |  |
| **Q43** | Dengue classification during the Reference Period by SINAN? | | | | | | | | Q43 |  |  |  |
|  | 1- Dengue without warning signs 3- Severe Dengue  2- Dengue with warning signs 4- another diagnosis | | | | | | | |  |  |  |  |
| Which laboratory exams confirmed a case of Dengue? | | | | | | | | | | |  |  |
| **Q44** | NS1: | | 1- Positive | | 2- Negative | | 3- Not permormed | | Q44 |  |  |  |
| **Q45** | IgG | | 1- Positive | | 2- Negative | | 3- Not permormed | | Q45 |  |  |  |
| **Q46** | IgM | | 1- Positive | | 2- Negative | | 3- Not permormed | | Q46 |  |  |  |
| **Q47** | Performed the RT-PCR exam?? | | | | |  |  |  | Q47 |  |  |  |
|  |  | | 1- Yes 2- No 9- Not applicable | | | | | |  |  |  |  |
| **Q48** | Which serotype was found? *(Write the serotype ex: DENV –1)* | | | | | | | | Q48 |  |  |  |
| **Q49** | Evolution of the case: | | | | | | | | Q49 |  |  |  |
|  | 1- Cure 2- death 3- death by other causes | | | | | | | |  |  |  |  |
| **FINANCIAL VARIABLES (DIRECT COSTS)** | | | | | | | | | | |  |  |
| **Q50-A** | Are you interviewing an outpatient case? 1-YES 2- NO | | | | | | | | **Q50-A** |  |  |  |
| **Q50** | How much was spent on doctor fees? (e.g. R$ 2000) | | | | | | | | **Q50** |  |  |  |
|  |  |  |  |  |  |  |  |  |  |  |  |  |
| **Q51** | How much was spent on laboratory exams? R$  *Obs. If there are extra fees from performing the exams, select.* | | | | | | | | **Q51** |  |  |  |
| **Q52** | How much was spent on imaging exams? (R$)  (include: X-ray, CAT scan, MRI, Ultrasound) | | | | | | | | **Q52** |  |  |  |
| **Q53** | How much was spent on food because of the disease? | | | | | | | | **Q53** |  |  |  |
| **Q54** | How much was spent on hosting? | | | | | | | | **Q54** |  |  |  |
| **Q54-A** | How much did you spend on medicine outside the hospital? | | | | | | | | **Q54-A** |  |  |  |
| **Q53** | How much was spend on hospital supplies? | | | | | | | | **Q53** |  |  |  |
| **Q54** | Spending on medicine at the Hospital or Health Centers. | | | | | | | | **Q54** |  |  |  |
| **Q55** | Expenses with various fees not covered by the health plan?  *(example: payment of consultation fees, etc.)* | | | | | | | | **Q55** |  |  |  |
| **Q56** | **Total value of all direct costs (sum of Q50 to Q55)** | | | | | | | | **Q56** |  |  |  |
| Q57 | Are you interviewing a hospitalized case? 1-YES 2-NO | | | | | | | | Q57 |  |  |  |
| **Q58** | What type of accommodations did the patient use? | | | | | | | | **Q58** |  |  |  |
|  | 1- Regular Ward | | |  | 3- Isolation | | 5- regular and ICU | |  |  |  |  |
|  | 2- plus room | | |  | 4- ICU |  | 9- Ignored | |  |  |  |  |
| **Q59** | Time hospitalized in infirmary, general admission, isolation? | | | | | | | | **Q59** |  |  |  |
| **Q60** | Time hospitalized in ICU? | | | | |  |  |  | **Q60** |  |  |  |
| **Q61** | Total hospitalization time (regular and ICU)? | | | | | |  |  | **Q61** |  |  |  |
| **Use of blood componentes** | | | | | | | | | | |  |  |
| **Q72** | Did the patient use blood products?? | | | | | | 1- Yes | 2- No | **Q72** |  |  |  |
| **Q72-A** | Prothrombin time (PT) and activated partial thromboplastin time (aPTT) are more than 1.5 times the standard reference values?  1-Yes 2-No | | | | | | | | **Q72-A** |  |  |  |
| **Q73** | **Red blood cells** | | | | |  | 1- Yes | 2- No | **Q73** |  |  |  |
| **Q74** | What is the Value of Hematocrit (%) when requested to transfuse? (write the value of the exam) | | | | | | | | **Q74** |  |  |  |
| **Q75** | What is the Value of Hemoglobin (g/100 mL) when requested to transfuse? (write the value of the exam) | | | | | | | | **Q75** |  |  |  |
| **Q75-A** | Were there significant bleeding events? (except gingival bleeding, epistaxis 1- Yes 2- No | | | | | | | | **Q75-A** |  |  |  |
| **Q75-B** | Signs of shock? 1- Yes 2- No | | | | | | | | **Q75-B** |  |  |  |
| **Q75-C** | Patient received adequate introvenous hydration prior to prescribing the bloodcomponent? 1- Yes 2- No | | | | | | | | **Q75-C** |  |  |  |
| **Q75-D** | What is the volume transfused? (in mL) | | | | | | | | **Q75-D** |  |  |  |
| **Q76** | **Were platelets used??** | | | |  |  | 1- Yes | 2- No | **Q76** |  |  |  |
| **Q77** | What was the platelets count when the transfusion was requested? (e.g. 400,000 por µL) | | | | | | | | **Q77** |  |  |  |
| **Q78** | Did patient show any active bleeding?  1- Yes 2- No | | | | | | | | **Q78** |  |  |  |
| **Q78-A** | Signs of shock? 1- Yes 2- No | | | | | | | | **Q78-A** |  |  |  |
| **Q78-B** | What was the volume of the infused platelets? (ml) | | | | | | | | **Q78-B** |  |  |  |
| **Q78-C** | Was Fresh Frozen Plasma (FFP) used? 1- Yes 2- No | | | | | | | | **Q78-C** |  |  |  |
| **Q79** | Developed a hospital infection while hospitalized?  1- Yes 2- No | | | | | | | | **Q79** |  |  |  |
| **Others information** | | | | | | | | | | |  |  |
| **Q80** | Thrombocytopenia ( platelets ≤ 20.000) | | | | | | 1-Yes | 2- No | **Q80** |  |  |  |
| **Q81** | Refused intake of liquid or food? | | | | | | 1-Yes | 2- No | **Q81** |  |  |  |
| **Q82** | Difficulty returning to the doctor in a medical condition that presents an immediate threat to their health. | | | | | | 1-Yes | 2- No | **Q82** |  |  |  |
| **Q83** | Comorbidity present? | | | | | | 1-Yes | 2- No | **Q83** |  |  |  |
| **Q84** | Severe organic impairment? | | | | | | 1-Yes | 2- No | **Q84** |  |  |  |
| **Q85** | Plasma leakage (e.g. pleural or pericardial effusions, ascites) | | | | | | 1-Yes | 2- No | **Q85** |  |  |  |
| **Q86** | Signs of altered tissue perfusion (e.g., altered skin perfusion, reduced urine output), convergence of blood pressure, decreased urinary volume, polish skin and cold sweats at extremities, lethargy, restlessness) | | | | | | 1-Yes | 2- No | **Q86** |  |  |  |
| **Q87** | Used blood products with criteria recommended by the WHO? | | | | | | 1-Yes | 2- No | **Q87** |  |  |  |
| **Q88** | Did not use blood products but exhibited criteria for use? | | | | | | 1-Yes | 2- No | **Q88** |  |  |  |

**Any notes about whether or not to use the blood component? Write here:**

**___________________________________________________________________________________________________________________________________________________________________________________________________________________________________________________________________________________________________________________________________________________________________________________________________________________________________________________________________________________________**
